# Comprehensive circRNA profiling of platelets and exosomes identifies hsa_circ_0061274 as a novel biomarker for lung adenocarcinoma

**DOI:** 10.3389/fimmu.2026.1761129

**Published:** 2026-03-18

**Authors:** Peiyin Zhang, Shan Liu, Bin Hu, Ping Leng, Zhuo Tang, Qun Yi, Dongsheng Wang, Yu Liu, Huaichao Luo, Feng Du, Sisi Yu

**Affiliations:** 1 Department of Clinical Laboratory, Sichuan Clinical Research Center for Cancer, Sichuan Cancer Hospital & Institute, Sichuan Cancer Center, University of Electronic Science and Technology of China, Chengdu, China; 2 Chongqing Key Laboratory of Sichuan-Chongqing Co-Construction for Diagnosis and Treatment of Infectious Diseases Integrated Traditional Chinese and Western Medicine, College of Medical Technology, Chengdu University of Traditional Chinese Medicine, Chengdu, China; 3 Yibin Hospital of the Affiliated Children's Hospital of Chongqing Medical University, Yibin, China; 4 Department of Thoracic Surgery, Sichuan Clinical Research Center for Cancer, Sichuan Cancer Hospital & Institute, Sichuan Cancer Center, Affiliated Cancer Hospital of University of Electronic Science and Technology of China, Chengdu, China; 5 Center for Natural Products, Chengdu Institution of Biology, Chinese Academy of Sciences, Chengdu, Sichuan, China; 6 Department of Critical Care Medicine, Sichuan Clinical Research Center for Cancer, Sichuan Cancer Hospital & Institute, Sichuan Cancer Center, University of Electronic Science and Technology of China, Chengdu, China; 7 Transfusion Medicine Research Center, The Institute of Blood Transfusion, Chinese Academy of Medical Sciences and Peking Union Medical College, Chengdu, China; 8 Department of Medical Oncology, Sichuan Clinical Research Center for Cancer, Sichuan Cancer Hospital & Institute, Sichuan Cancer Center, University of Electronic Science and Technology of China, Chengdu, China

**Keywords:** circRNA, hsa_circ_0061274, liquid biopsy, lung adenocarcinoma, platelet

## Abstract

**Background:**

Lung adenocarcinoma (LUAD) is usually detected late; sensitive, minimally invasive early-detection tools are urgently needed. Circular RNAs (circRNAs) in liquid biopsies are promising cancer biomarkers, yet it remains unclear which blood component—platelets or plasma exosomes—offers the richest and most informative circRNA source.

**Materials and methods:**

High-throughput RNA sequencing was performed on platelets and plasma exosomes collected from LUAD patients and age-matched healthy donors. Differential circRNAs expression was analyzed after stringent quality filtering and normalization. A candidate reference gene was selected by stability testing (geNorm, NormFinder); diagnostic performance of the top LUAD-associated circRNA was validated in independent cohorts encompassing healthy controls and patients with benign pulmonary nodules; ROC curves were generated and AUCs calculated.

**Results:**

Platelets contained 15–20-fold more distinct circRNAs than plasma exosomes. hsa_circ_0001380 was identified as a stably expressed reference suitable for platelet circRNA quantification. hsa_circ_0061274 was significantly down-regulated in LUAD platelets (log2FC ≈ −1.8, FDR < 0.01). In validation cohorts, hsa_circ_0061274 discriminated LUAD from healthy controls with an AUC of 0.85 (95% CI 0.79–0.91), from benign pulmonary nodules with an AUC of 0.75 (95% CI 0.68–0.82), and stage I LUAD with an Area Under the Curve (AUC) of 0.68 (95% CI 0.60–0.76). Platelet-derived circRNAs thus provide robust, minimally invasive biomarkers for early LUAD detection and pulmonary nodule characterization.

## 1 Introduction

Cancer constitutes a major global health threat, with both incidence and mortality rising annually among individuals aged 30–69 years (1, 2). Early-stage lung cancer is often clinically silent, consequently, the majority of cases are diagnosed at an advanced stage, for which the 5-year survival rate remains only ~20% (3–5). Lung cancer is broadly classified into non-small-cell lung cancer (NSCLC) and small-cell lung cancer (SCLC), with NSCLC accounting for ~85% of all cases (6–8). LUAD is the most common subtype, representing approximately 40% of lung cancers, while LUSC comprises 25–30% (9–11).

Current diagnostic strategies for lung cancer rely primarily on two approaches. The first is population-based screening, for which low-dose computed tomography is the most effective modality (12). Nevertheless, imaging alone cannot definitively diagnose lung cancer; histopathological confirmation of lesional tissue remains the gold standard (13). Tissue acquisition, however, is invasive, costly, and necessitates serial follow-up imaging, imposing physiological, psychological on patients. Moreover, a single biopsy specimen may not fully capture tumor heterogeneity or permit real-time monitoring of clonal evolution. The invasive procedure itself may even increase the risk of tumor cell dissemination, ultimately compromising survival and prognosis (14).

Liquid biopsy offers several advantages: specimens are easily obtained, can provide a systemic portrait of tumor heterogeneity, and enable real-time monitoring of disease evolution (15). The principal liquid biopsy analytes include exosomes, circulating tumor cells (CTCs), tumor-educated platelets (TEPs), circulating tumor DNA (ctDNA), microRNAs (miRNAs), circular RNAs (circRNAs), extracellular vesicles (EVs), and circulating tumor-derived vascular endothelial cells (CTECs) (16, 17). ctDNA comprises short fragments of tumor-derived DNA released into the circulation. Several studies have shown that plasma ctDNA levels are significantly higher in patients with non-small-cell lung cancer (NSCLC) than in healthy controls or in individuals with chronic respiratory diseases (18). Integrating ctDNA mutational profiling with DNA-methylation signatures can accurately discriminate lung cancer from benign nodules. Notably, this combined approach performs better in stage IB than in stage IA disease, opening new avenues for the early detection of lung cancer (19). Nevertheless, technical hurdles—including limited analytical sensitivity, high cost, prolonged turnaround times, and complex workflows—currently restrict the widespread clinical adoption of ctDNA assays.

Circulating tumor cells (CTCs) are intact tumor cells that have detached from the primary tumor or metastatic deposits and entered the bloodstream. Their capture is pivotal for early detection, disease monitoring, prognostication, and mechanistic studies of metastasis (20–23). The CellSearch^®^ system is currently the only FDA-approved platform for CTC enumeration. Recognizing the analytical potential of CTCs, several research groups have developed the Chimergx^®^-i120 platform, which employs machine-learning algorithms to enrich, label, and characterize CTCs with high precision. Clinical studies have validated Chimergx^®^-i120 as a robust tool for CTC detection and downstream genomic analyses (24).

Platelets are anucleate cytoplasmic fragments derived from bone marrow megakaryocytes (25). Beyond their canonical hemostatic functions, platelets are increasingly recognized as active participants in the pathogenesis of numerous diseases, most notably cancer (25, 26). Platelets are also increasingly recognized as key modulators of innate and adaptive immunity. They express functional immune receptors and store numerous immunoregulatory cytokines in their granules, enabling them to actively recruit leukocytes, modulate NK cell and T cell functions, and shape the tumor immune microenvironment. Although platelets are small and lack a nucleus, they harbor a remarkably diverse molecular repertoire, including functional ribosomes, signaling proteins, and multiple RNA species [messenger RNA (mRNA), microRNA (miRNA), and circular RNA (circRNA)] (27). Tumor cells secrete a spectrum of bioactive molecules [e.g., proteases, tissue factor, adenosine diphosphate (ADP), matrix metalloproteinases, and thromboxane A2 (TXA2)] that engage platelet receptors. These effectors may be released directly into the circulation or packaged within tumor-derived exosomes, which can also transfer tumor RNA to platelets (28). Tumor-induced signaling culminates in measurable alterations in platelet RNA content, achieved through modulation of protein translation followed by RNA decay, stimulation of specific splicing events, or sequestration and release of circulating RNA (29, 30). Notably, such tumor-driven RNA alterations extend to circular RNAs (circRNAs), which are enriched in platelets and may serve as functional regulators or biomarkers of tumor–platelet crosstalk (31, 32). For instance, the downregulation of hsa_circ_0061274 observed in our study likely reflects a specific aspect of this TEP reprogramming, although its exact mechanistic role—whether as a miRNA sponge, a protein scaffold, or a modulator of platelet activation—remains to be elucidated. Collectively, these tumor-educated transcriptomic changes define the concept of “tumor-educated platelets” (TEPs) (33). Importantly, TEPs not only serve as passive sensors of tumor presence, but also actively participate in immune evasion by transferring regulatory molecules to immune cells and by altering their own immunomodulatory phenotype (34, 35). Therefore, platelet-derived circRNAs may offer a unique window into the dynamic interplay between the tumor and the host immune system.

Platelet-based liquid biopsy has rapidly evolved into a focal point of translational oncology research (36). Through comparative RNA Sequencing (RNA-seq) of platelets from healthy donors and metastatic lung cancer patients, a landmark study identified 5,003 differentially expressed transcripts in tumor-educated platelets (TEPs) (37). Employing the ThromboSeq algorithm, the investigators distinguished cancer patients from controls with an accuracy of 96%, providing robust evidence that platelet RNA signatures can facilitate early detection and precision management of malignancy (38). Importantly, stage-stratified platelet RNA profiling not only identified early-stage lung cancer but also discriminated lung adenocarcinoma (LUAD) from squamous-cell carcinoma (LUSC) (39).

High-throughput sequencing of blood-derived RNA has revealed that circRNAs—covalently closed, single-stranded non-coding RNAs generated by back-splicing—are markedly enriched in whole blood compared with serum or plasma. Lacking 5′ caps and 3′ poly(A) tails, circRNAs are highly resistant to exonucleases, conferring exceptional stability and inter-individual conservation (40). These properties render circRNAs attractive candidates for diagnostic and therapeutic exploitation in oncology (41, 42).

Exosomes—the smallest class of extracellular vesicles—transport bioactive cargo (proteins, nucleic acids, lipids, and metabolites) that modulate immunity, tumor metabolism, and drug resistance (43–45). Plasma exosomal circRNAs have shown diagnostic promise in multiple cancers: a panel of hsa_circ_0001439, hsa_circ_0001492, and hsa_circ_0000896 distinguished LUAD from controls with an AUC of 0.805 (46); hsa_circ_0055202, hsa_circ_0074920, and hsa_circ_0043722 were up-regulated in exosomes from glioblastoma patients (47).

Despite these encouraging data, exosome isolation remains problematic. Ultracentrifugation, the current gold standard, causes >80% loss of vesicles and frequently disrupts their lipid bilayers, compromising downstream analyses (48). Platelets, in contrast, are far more readily purified and contain circRNA at levels exceeding those of nucleated tissues by an order of magnitude; exon content is 12.7-fold higher than in corresponding tissues (49). Thomas et al. demonstrated that circNRIP1 is significantly downregulated in NSCLC platelets, validating the diagnostic potential of platelet circRNAs (50). Building on this work, the same group employed a machine-learning framework integrating only five features—two circRNAs (circSLC8A1, circCHD9) and three mRNAs (PSMB9, RUNX1, LILRB1)—to classify early-stage NSCLC versus controls with an AUC of 0.96 ± 0.03 and an accuracy of 86% (31).

To date, few studies have simultaneously interrogated platelet and plasma exosomal circRNAs within the same patient, and direct comparisons of their diagnostic performance are lacking. Moreover, LUAD-specific platelet circRNA signatures—and their capacity to discriminate benign versus malignant pulmonary nodules—remain largely unexplored. In the present study, by sequencing paired platelets and plasma exosomes from LUAD patients and controls, we identified compartment-specific circRNA signatures, compared their diagnostic performance, and developed a platelet-based assay for early LUAD detection and nodule profiling.

## 2 Methods

### 2.1 Study participants

This study is a retrospective study utilizing anonymized archived samples and data. It has been approved by the Ethics Committee of Sichuan Cancer Hospital (approval number: SCCHEC-02-2020-043), exempting the need to obtain individual informed consent from patients, Participants were selected based on strict inclusion and exclusion criteria to ensure representativeness and reliability of the results. Participants were recruited from the Department of Thoracic Surgery at Sichuan Cancer Hospital between January 2022 and December 2023. Patients in the early-stage lung adenocarcinoma (LUAD) group were enrolled based on the following criteria: age between 18 and 80 years, an initial diagnosis by LDCT and subsequent pathological confirmation of stage I disease according to the 8th edition TNM staging system, a single primary tumor with no history of other malignancies, no bleeding disorders related to platelet count or function, and no use of aspirin-like medications or procedures affecting platelets within two weeks prior to blood sampling. Healthy donors (HDs) were enrolled based on the following criteria: age 18–80 years, a body mass index (BMI) between 19 and 24 kg/m², normal or clinically non-significant results on routine blood tests, biochemistry, liver/kidney function tests, physical examination, echocardiography, and electrocardiogram, no history of chronic or serious illnesses, and no pulmonary nodules detected on LDCT screening. Specifically, healthy donors were defined as individuals with no history of smoking, no known comorbidities, and no acute or chronic inflammatory conditions at enrollment. Exclusion criteria applied to both groups included age <18 or >80 years, presence of other chronic diseases or malignancies, any prior anticancer therapy before diagnosis for LUAD patients, any previous treatment that could affect platelet function, and any other condition considered unsuitable for participation by the investigators, the benign pulmonary nodule (BPN) control group comprised patients with histopathologically confirmed hamartomas, granulomas, and organizing pneumonia. The sample source of the cohort of luad patients and healthy donors analyzed in this study is the same as our previous study focusing on miRNA markers, DOI: 10.3389/fimmu.2025.1619448. All procedures were performed in accordance with the ethical standards of the institutional research committee and with the 1964 Declaration of Helsinki and its later amendments. Demographic and clinical variables were systematically documented for subsequent analyses.

### 2.2 Platelet and plasma exosome isolation and purity assessment

**Figure 1:**
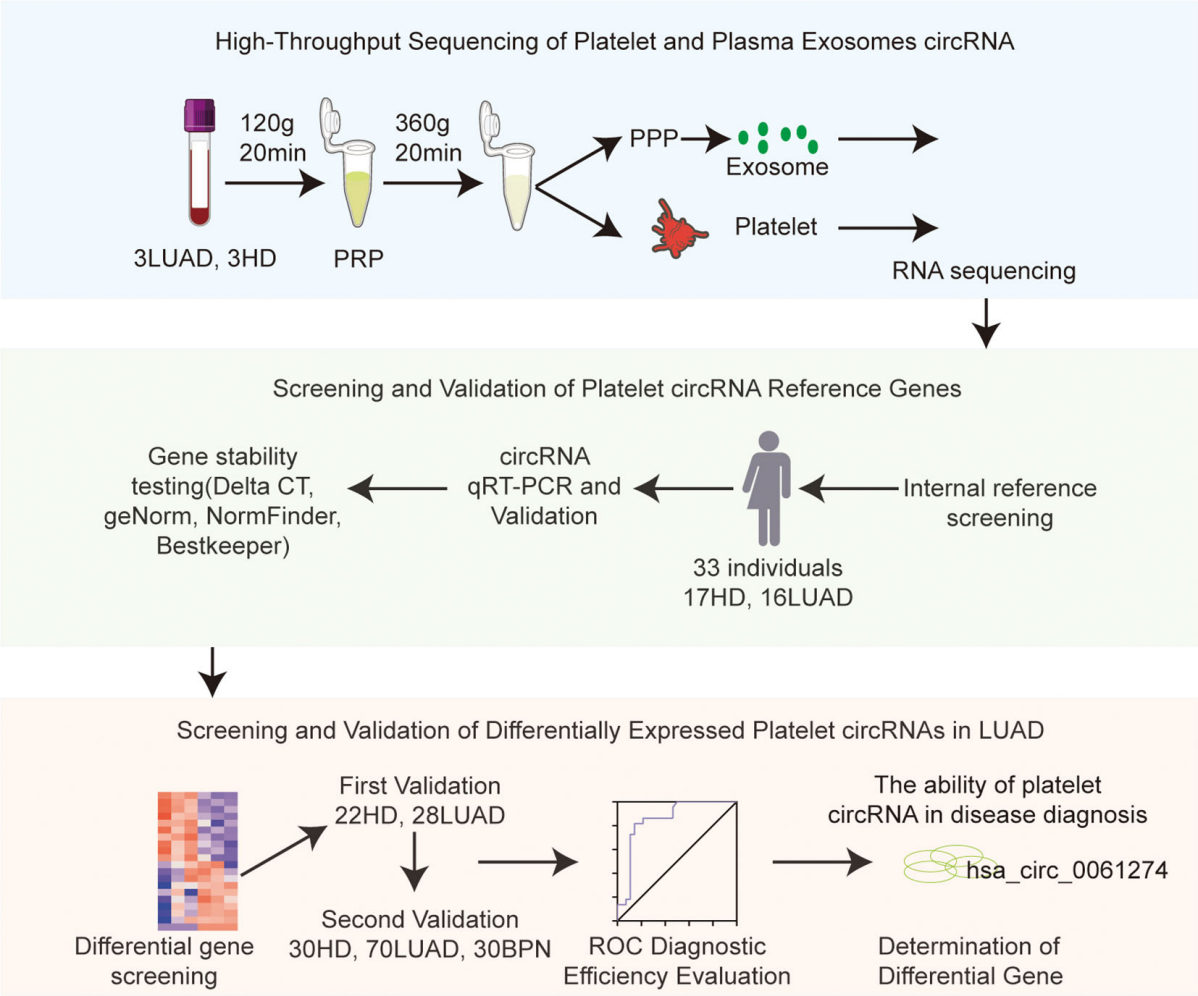
Flowchart of the experimental workflow.

Platelets were isolated from whole blood collected in EDTA anticoagulant vacuum tubes. A total of 2 mL of venous blood was subjected to low-speed centrifugation at 120 g for 20 minutes to separate cellular components, yielding a platelet-rich plasma (PRP) layer. The PRP was transferred to a new tube and centrifuged again at 360 g for 20 minutes to obtain platelet pellets (37, 51). Purity was assessed using an automated blood cell analyzer, ensuring no more than five nucleated cells per ten million platelets. In this study, we used size exclusion chromatography (SEC) to separate plasma exosomes. Based on the principle of molecular sieve, the method uses a chromatographic column filled with porous gel particles to achieve separation according to the difference of hydrodynamic diameters of different components: the exosomes (with a diameter of about 30–150 nm) cannot enter the gel pore due to their large volume, and flow rapidly through the gap between particles driven by the mobile phase; However, soluble proteins, lipoproteins and small molecular impurities in plasma enter the gel pore and remain due to the extension of the path, thus effectively separating from the exosomes. This protocol has been validated to yield exosomes with typical characteristics (52). Due to limitations in sample availability, direct biophysical characterization of the isolated exosome preparation was not performed in this particular experiment (**Figure 1**).

### 2.3 Platelet and exosome RNA sequencing

Total RNA was extracted from the isolated platelets and plasma exosomes using a rapid RNA extraction kit. The quality of the extracted RNA was assessed using a NanoDrop 2000 spectrophotometer, ensuring an OD260/OD280 ratio between 1.8 and 2.0 to confirm purity. For circRNA sequencing, we adopted a ribosomal RNA depletion approach using Ribo-Zero Gold reagents to remove rRNA, followed by standard RNA-seq library construction. We acknowledge that this method does not specifically enrich for circRNAs and may have lower sensitivity for low-abundance circRNAs; however, it remains suitable for comparative profiling of abundant circRNAs as performed in this discovery-phase study. The constructed cDNA libraries were sequenced on the Illumina NovaSeq 6000 platform. Bioinformatic analysis of circRNAs was performed as follows: Sequencing reads were aligned to the human reference genome GRCh38/hg38 using BWA-MEM. CircRNAs were identified using CIRI (version 2) with default parameters. Only circRNAs supported by at least two independent back-splicing junction reads were retained. Known circRNAs were annotated using the circBase database.

### 2.4 Quantification analysis of circRNAs

Quantification of circRNAs was performed by qRT−PCR. Briefly, target sequences were retrieved from circBase, and all forward and reverse primers were designed to span the unique back−splice junction of each circRNA to ensure specific amplification of the circular isoform. For key candidate circRNAs (including the internal control and the biomarker hsa_circ_0061274), the specificity of the junction−spanning primers was empirically confirmed by Sanger sequencing of the PCR amplicons. Total RNA was extracted from platelets using a rapid extraction kit, treated with gDNA Eraser (Takara) to remove genomic DNA, and reverse−transcribed into cDNA using the PrimeScript RT Reagent Kit (Takara). Quantitative real−time PCR was then carried out using TB Green Premix Ex Taq II (Takara) on an MA−6000 system. Each sample was run in triplicate under the following conditions: 95°C for 30 s, followed by 40 cycles of 95°C for 5 s and 60°C for 30 s; melt−curve analysis was performed after each run to verify amplicon specificity.

### 2.5 Analysis and screening of reference circRNAs and differential circRNAs

Raw sequencing reads were preprocessed, which included quality control, adapter trimming, and alignment to the human reference genome (GRCh38/hg38) using BWA-MEM. CircRNAs were identified and quantified from the alignments using CIRI (Section 2.3). The resulting circRNA read counts were used as input for differential expression analysis with the R package DESeq2, which applies an internal median-of-ratios normalization. CircRNAs with |log_2_FoldChange| ≥ 0.5 and an adjusted P-value (FDR) < 0.05 were considered significantly differentially expressed. From the differentially expressed circRNAs, downregulated candidates were selected for validation. Concurrently, candidate reference circRNAs were evaluated for stable expression across all cohorts (HD, LUAD, BPN) using four stability algorithms (Delta Ct, geNorm, NormFinder, BestKeeper). This analysis identified hsa_circ_0001380 as the optimal endogenous control for subsequent qRT-PCR normalization and validation studies.

### 2.6 The analysis of stability and diagnostic value for platelet circRNAs

In this study, the stability of candidate reference circRNAs was evaluated using four algorithms (Delta Ct, geNorm, NormFinder, and BestKeeper). Results indicated that hsa_circ_0001380 exhibited the highest stability, making it suitable as a reference gene. Subsequently, ROC curve analysis was performed to assess the diagnostic value of platelet circRNAs, particularly in distinguishing LUAD from benign pulmonary nodules. The area under the curve (AUC) was calculated to quantify diagnostic performance.

## 3 Results

### 3.1 Comparative profiling of circRNA landscapes in platelets and plasma exosomes

**Figure 2:**
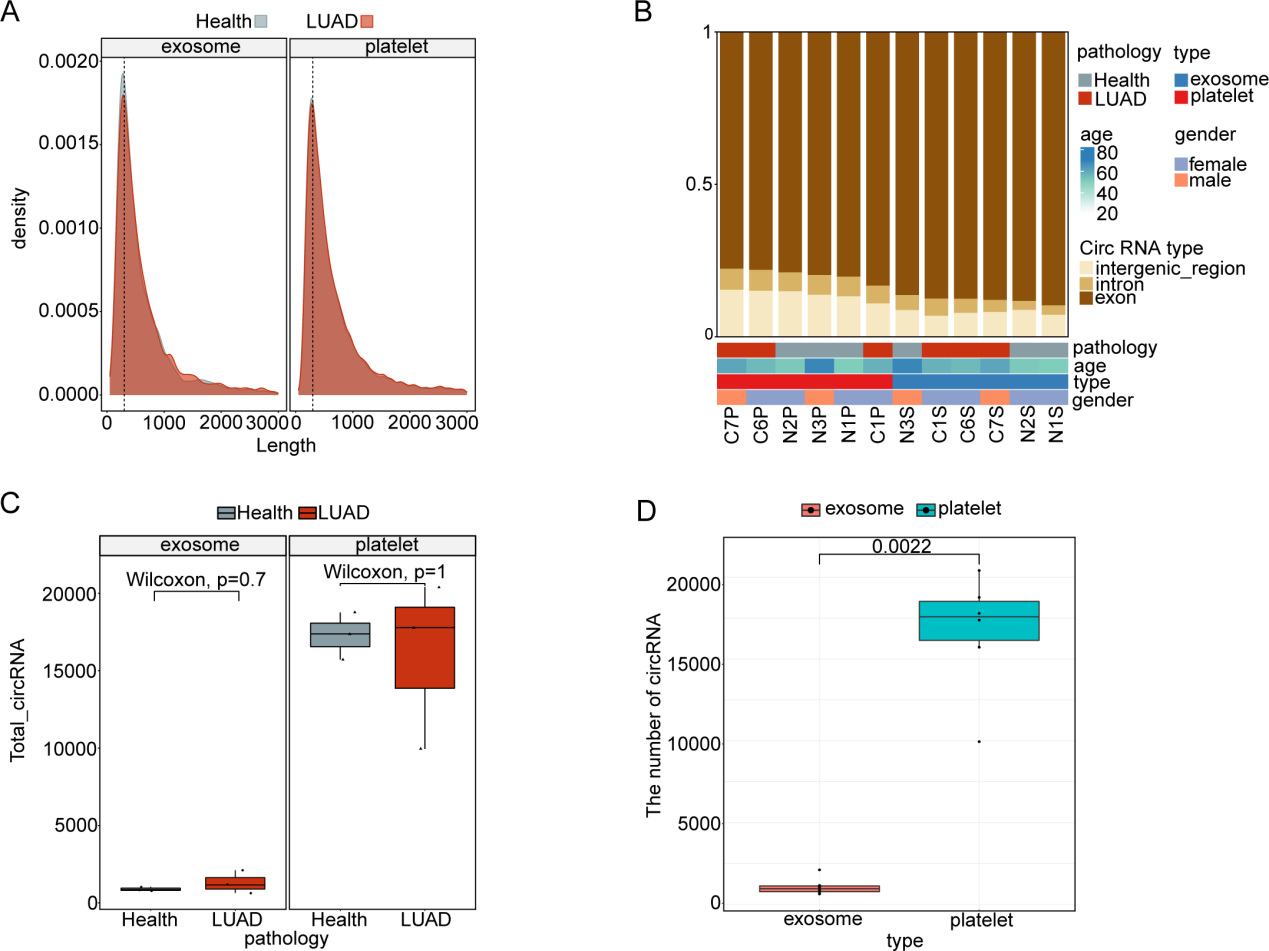
General features of platelet and exosome sequencing. **(A)** The length of circRNA between platelets and plasma exosomes. **(B)** Annotation of circRNA classification between platelets and plasma exosomes. **(C)** Differences in the number of circRNAs screened for plasma (P = 0.7) and platelet (P = 1) exosomes of HD and LUAD. **(D)** Differences in the number of circRNAs screened from platelet and plasma exosomes.

High-throughput circRNA sequencing was performed on matched platelet and plasma exosome samples from three untreated LUAD patients (stages IA-IB) and three healthy donors (**Table 1**), the detailed circRNA count and length metrics for each sample are provided in **Supplementary Tables 1**–**5**. The results revealed a similar length distribution of circRNAs in platelets and exosomes, primarily spanning 100–400 nucleotides, with no significant differences observed (**Figure 2A**). Both platelets and exosomes were highly enriched in exon-derived circular RNAs (>95% of total circRNAs), with no significant differences in compositional distribution between the LUAD and healthy groups (**Figure 2B**).

**Table 1:** Basic clinical information about the participants.

Sample	Group	Gender	Age	Smoking	Stage	T	N	M
C1	LUAD	Female	57	No	IA	1	0	0
C2	LUAD	Female	55	No	IA	1	0	0
C3	LUAD	Male	60	Yes	IB	2	0	0
N1	HD	Female	50	No	/	/	/	/
N2	HD	Female	51	No	/	/	/	/
N3	HD	Male	68	Yes	/	/	/	/

Regarding circRNA abundance, no significant differences were identified between the LUAD and healthy groups in either platelets or exosomes (**Figure 2C**). However, a noteworthy finding was that platelets exhibited substantially higher circRNA levels compared to plasma exosomes. Specifically, 15,000-20,000 circRNAs were detected per platelet sample, whereas approximately 1,000 circRNAs were detected per exosome sample (P = 0.0022) (**Figures 2C, D**). This 15- to 20-fold enrichment was consistently observed across all six subjects, representing the first direct quantitative comparison of circRNA abundance between platelets and exosomes within the same individuals. These findings suggest that platelets harbor a significantly larger and more diverse reservoir of circRNAs compared to plasma exosomes, highlighting their potential as a robust source for biomarker discovery. It is noteworthy that the current discovery-phase sequencing analysis involved a relatively small sample size, which was intended for preliminary head-to-head comparisons and candidate molecule screening. Independent validation in larger cohorts will be required to confirm the generalizability of these findings.

### 3.2 Screening of reference genes for platelet circRNA

**Figure 3:**
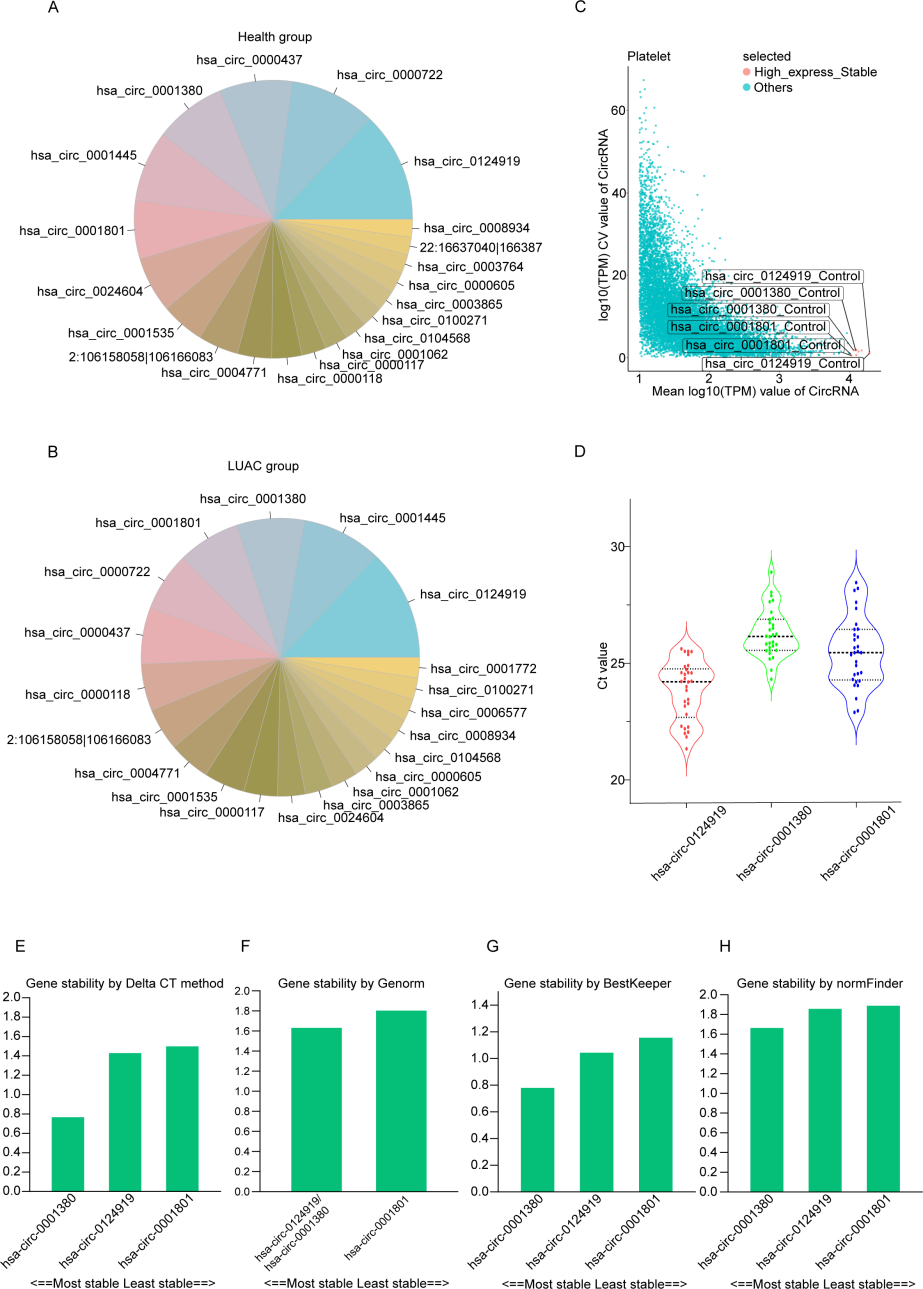
Identification and validation of reference circRNAs in platelets. **(A)** Ranking of the 20 most highly expressed circRNAs in healthy donors (HD). **(B)** Ranking of the 20 most highly expressed circRNAs in lung adenocarcinoma (LUAD). **(C)** Three platelet circRNA candidate internal reference genes with high and stable expression level were detected in the platelets of the control group and lung cancer group. **(D)** Distribution of CT values of platelet three candidate reference genes in all samples. **(E–H)** The expression stability ranking diagram of the three candidate genes in the four algorithms.

This section includes 33 subjects, comprising 17 healthy donors (HD) and 16 lung adenocarcinoma (LUAD) patients. The two groups are comparable in terms of gender (**Table 2**), and there is no statistical difference in age. Based on platelet RNA-seq data, we identified the top 20 most highly expressed circRNAs in both the healthy donor and LUAD groups (**Figures 3A, B**), detailed data are available in **Supplementary Table 6**. According to pre-established screening criteria, which included a high expression level (log_10_(TPM) ≥ 4) and low variability (coefficient of variation < 20%) across groups, we selected three stable and highly expressed circRNAs as candidate reference genes: hsa_circ_0124919, hsa_circ_0001380, and hsa_circ_0001801 (**Figures 3C, D**), detailed data are available in **Supplementary Table 7**. All three candidates ranked among the top 20 most abundant circRNAs in both HD and LUAD samples.

**Table 2:** Characteristics of all enrolled subjects for validation of candidate reference CircRNAs.

Characteristics	Total	HD	LUAD	P value
Gender				
Male	11 (33.33%)	5 (45.45%)	6 (54.55%)	0.695
Female	22 (66.67%)	12 (54.55%)	10 (45.45%)	/
Age group				
≤55	15 (45.45%)	6 (40.00%)	9 (60.00%)	0.528
>55 and ≤80	18 (54.55%)	11 (61.11%)	7 (38.89%)	/
Median (IQR)	57.0 (49.0-64.0)	59.0 (52.0-65.0)	56.0 (48.0-62.0)	0.325
Stage				
I-II	10 (62.50%)	/	10 (62.50%)	/
III-IV	4 (25.00%)	/	4 (25.00%)	/
NA	2 (12.50%)	/	2 (12.50%)	/

Using the comparative ΔCt method, we assessed the stability of each candidate by comparing pairwise variation between samples. The analysis revealed that hsa_circ_0001380 exhibited the smallest standard deviation (SD = 1.571), indicating the highest stability among the candidates (**Figure 3E**), detailed data are available in **Supplementary Table 8**. The geNorm algorithm further supported the stability of hsa_circ_0001380, showing it to be among the most stable genes, along with hsa_circ_0124919 (**Figure 3F**). Consistent results were obtained using the BestKeeper and NormFinder algorithms, both of which identified hsa_circ_0001380 as the most stable reference gene (**Figures 3G, H**). Comprehensive ranking based on the four algorithms confirmed that hsa_circ_0001380 is the most stable reference circRNA in platelets for normalizing gene expression studies in LUAD (**Table 3**).

**Table 3:** Ranking of the stability of the three reference genes.

Methods	Reference genes stability value rank		
	First	Second	Third
Delta Ct	hsa_circ_0001380	hsa_circ_0124919	hsa_circ_0001801
Genorm	hsa_circ_0001380 hsa_circ_0124919	hsa_circ_0001801	/
Normfinder	hsa_circ_0001380	hsa_circ_0124919	hsa_circ_0001801
Bestkeeper	hsa_circ_0001380	hsa_circ_0124919	hsa_circ_0001801
Recommended comprehensive ranking	hsa_circ_0001380	hsa_circ_0124919	hsa_circ_0001801

### 3.3 Screening and validation of platelet differential circRNAs in lung adenocarcinoma

**Figure 4:**
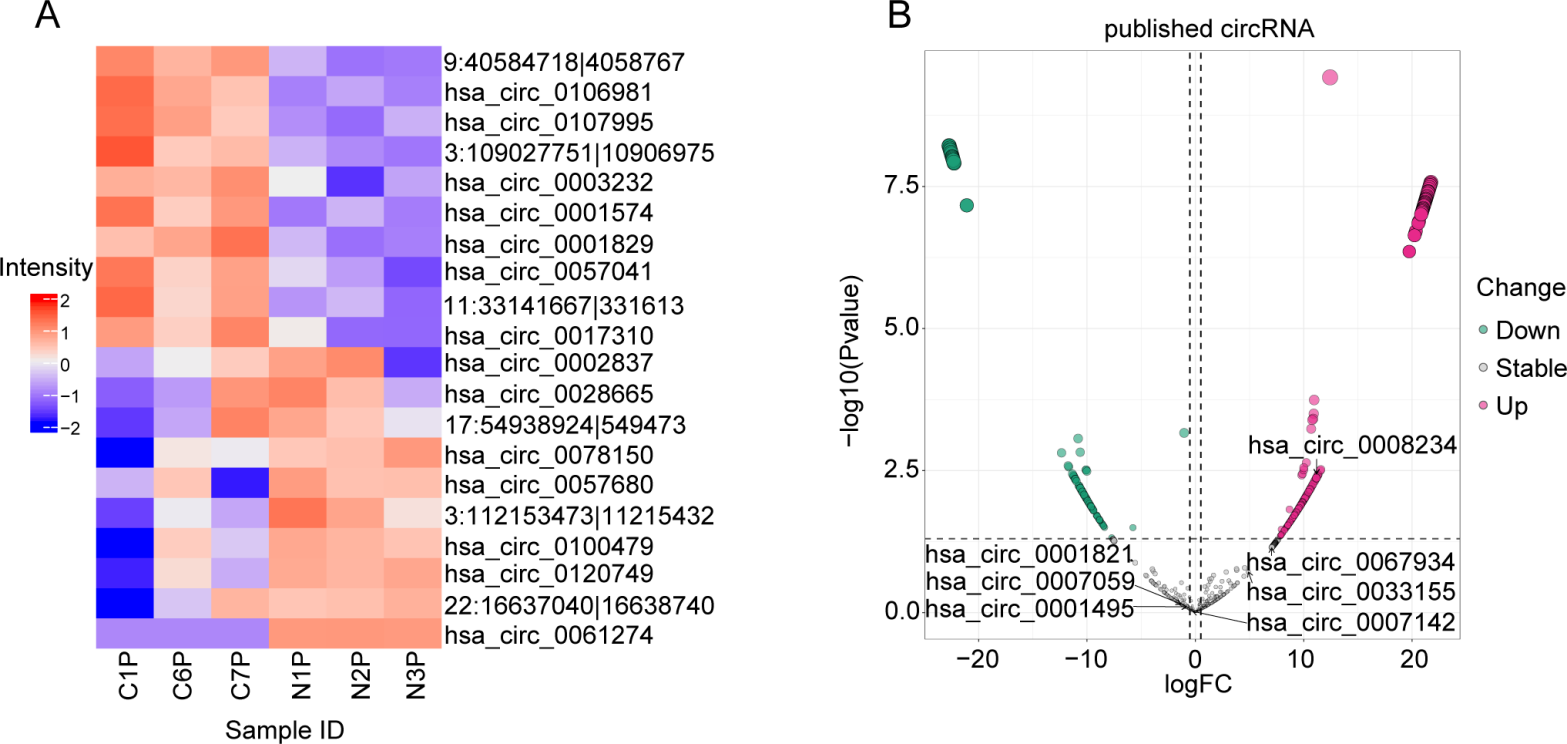
Screening of platelet differential circRNAs. **(A)** Heat map analysis of platelet differential circRNAs. **(B)** Cyclic RNAs that have been reported to be altered in diseases.

Differentially expressed platelet circRNAs were analyzed and screened using the R package DESeq2. The selection of differentially expressed circRNAs was based on fold change and P-values to evaluate the significance of differences, with thresholds set at |log_2_FoldChange| ≥ 0.5 and P < 0.05. The top 10 upregulated and downregulated circRNAs in platelets are illustrated (**Figure 4A**), detailed data are available in **Supplementary Table 9**. In addition, some published circrnas that have changed in diseases have also been presented (**Figure 4B**) (53–59). These differential circRNAs reveal significant changes in the expression profiles between healthy donors and LUAD patients, providing new insights into the molecular mechanisms underlying LUAD. Among these circRNAs, hsa_circ_0001380 was identified as the most significantly downregulated circRNA, suggesting its potential as a differential biomarker. Consequently, it was selected for subsequent functional validation.

### 3.4 Preliminary validation of differential circRNAs by RT-qPCR

**Figure 5:**
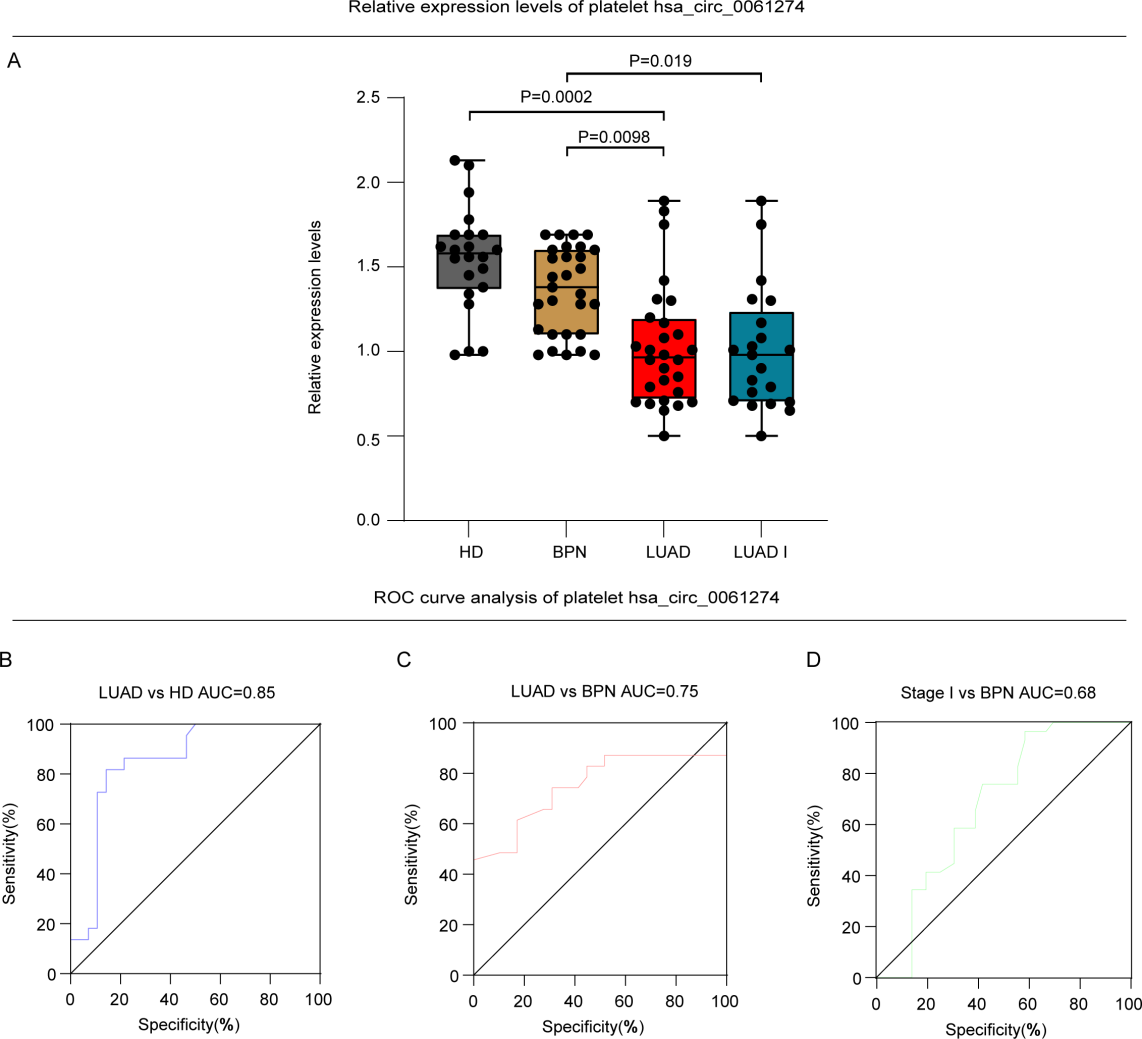
Analysis of platelet hsa_circ_0061274 expression and ROC curve in HD, LUAD and BPN groups. **(A)** Relative expression level of platelets hsa_circ_0061274 in between HD and LUAD (P = 0.0002), LUAD and BPN (P = 0.0098), and between LUAD I and BPN (P = 0.019). **(D–F)** The ability of platelet hsa_circ_0061274 for diagnosis of HD and LUAD (AUC = 0.85), diagnosis of LUAD and BPN (AUC = 0.75), diagnosis of LUAD I and BPN (AUC = 0.68).

Among the circRNAs downregulated in platelets of lung adenocarcinoma (LUAD) patients, hsa_circ_0061274 displayed the highest abundance. Although its downregulation has been documented in gastric cancer (60), this alteration remains uncharacterized in LUAD. Therefore, we performed an initial validation using platelets from 22 healthy donors (HD) and 28 untreated LUAD patients (**Table 4**). The two cohorts were well matched for sex, age, and platelet parameters (all P > 0.05) (**Table 4**). qRT-PCR confirmed that hsa_circ_0061274 was markedly reduced in LUAD platelets relative to HD controls (P = 0.0002) (**Figure 5A**). Thus, we identified hsa_circ_0061274 as a potential differential gene and will proceed with further large-scale validation.

**Table 4:** Clinical characteristics of all participants.

Characteristics	Total	First validation	Second validation
Gender			
Male	76 (42.22%)	18 (36.0%)	58 (44.62%)
Female	104 (57.78%)	32 (64.0%)	72 (55.38%)
Age group			
Median (IQR)	54.0 (47.0–63.0)	55.0 (49.0–62.0)	53.0 (46.0–64.0)
Histological classification			
LUAD	98 (54.44%)	28 (56.0%)	70 (53.85%)
BNP	30 (16.67%)	/	30 (23.08%)
HD	52 (28.89%)	22 (44.0%)	30 (23.07%)
Stage			
I	48 (48.98%)	12 (42.86%)	36 (51.43%)
II	20 (20.41%)	8 (28.57%)	12 (17.14%)
III	10 (10.20%)	2 (7.14%)	8 (11.43%)
IV	9 (9.18%)	3 (10.71%)	6 (8.57%)
NA	11 (11.23%)	3 (10.72%)	8 (11.43%)

### 3.5 Further validation of platelet hsa_circ_0061274 by RT-qPCR

To further investigate the relationship between hsa_circ_0061274 expression in platelets and the nature of pulmonary nodules, we conducted a secondary validation. A total of 130 participants were included in this validation, comprising 70 lung adenocarcinoma (LUAD) patients, 30 healthy donors (HD) and 30 benign pulmonary nodule (BPN) patients. There were no statistically significant differences in sex or age between the two groups (**Table 4**).

The qRT-PCR results indicated that the expression level of hsa_circ_0061274 in platelets was significantly lower in the LUAD group compared to the BPN group (P = 0.0098) (**Figure 5A**). Furthermore, we examined the relative expression of hsa_circ_0061274 in platelets between the stage I LUAD group (characterized by a single lesion smaller than 3 cm) and the BPN group. Remarkably, the results revealed that the expression of hsa_circ_0061274 in the stage I LUAD group was also significantly lower than in the BPN group (P = 0.019) (**Figure 5A**). These findings provide important insights into the potential application of hsa_circ_0061274 in the early diagnosis of lung cancer. The differential expression of platelet hsa_circ_0061274 in diagnosing healthy donors, LUAD, and BPN suggests its promise as a potential biomarker for distinguishing malignant from benign pulmonary nodules.

### 3.6 Diagnostic value of platelet hsa_circ_0061274

To further validate the diagnostic value of platelet circRNA hsa_circ_0061274 in lung adenocarcinoma (LUAD), we conducted a receiver operating characteristic (ROC) analysis to assess its diagnostic performance. The results showed that when distinguishing LUAD patients from healthy donors (HD), the area under the curve (AUC) for hsa_circ_0061274 was 0.85 (95% CI: 0.740-0.963), with a sensitivity of 85.70% and a specificity of 81.80% (P < 0.001) (**Figure 5B**), demonstrating high diagnostic accuracy. In differentiating malignant pulmonary nodules from benign pulmonary nodules (BPN), the AUC value was 0.75 (95% CI: 0.688-0.939), with a sensitivity of 78.60% and a specificity of 77.30% (P < 0.001) (**Figure 5C**), indicating significant diagnostic capability. Notably, in the early (stage I) LUAD subgroup, which included only single lesions with a maximum diameter of <3 cm, hsa_circ_0061274 still demonstrated an AUC of 0.68 (95% CI: 0.623-0.895) in distinguishing early LUAD from BPN, with a sensitivity of 50.00% and a specificity of 95.50% (P = 0.002) (**Figure 5D**, **Table 5**).

**Table 5:** Evaluation of diagnostic efficacy of hsa_circ_0061274 using ROC analysis.

Group	AUC (95%CI)	Se (%)	Sp (%)	*P*
LUAD vs HD	0.85 (0.740 to 0.963)	85.70	81.80	<0.001
LUAD vs BPN	0.75 (0.688 to 0.939)	78.60	77.30	<0.001
LUAD stage I vs BPN	0.68 (0.623 to 0.895)	50.00	95.50	0.002

These findings suggest that hsa_circ_0061274 holds promise as a biomarker for the early screening and assessment of malignancy in pulmonary nodules, further supporting its importance in clinical applications.

## 4 Discussion

Platelets, traditionally viewed as anucleate cell fragments central to hemostasis, are now recognized as a dynamic reservoir of functional RNAs. In liquid-biopsy research they are attractive because they are abundant, easily isolated, contain high-quality RNA, and rapidly respond to tumor-derived signals (61, 62). Here we demonstrate that platelets provide a far richer and more stable source of circular RNAs (circRNAs) than plasma exosomes, containing >15-fold more circRNA copies. Exploiting this advantage, we performed platelet circRNA sequencing and validation, and—for the first time—established platelet hsa_circ_0061274 as a single-molecule classifier that discriminates early-stage lung adenocarcinoma (LUAD) from benign pulmonary nodules and from healthy individuals without any enzymatic amplification or multi-gene panel. Importantly, the marker also separates benign nodules from stage I LUAD.

CircRNAs are a unique class of non-coding RNAs generated by non-canonical back-splicing, forming covalently closed loops (63, 64). Their abundance correlates with cancer type, stage, and other clinical variables, and numerous circRNAs have been proposed as diagnostic or prognostic biomarkers for cancer screening and surveillance (65, 66). Exosomes are nano-sized extracellular vesicles secreted into bio-fluids such as blood, urine, and cerebrospinal fluid (67, 68). Exosomal circRNAs modulate cancer progression, and differential expression of plasma exosomal circRNAs has shown diagnostic, monitoring, and prognostic value (68, 69). However, the lack of standardized isolation and analysis protocols, time-consuming procedures, high cost, limited storage stability, low yield, sub-optimal purity, and poor target-enrichment efficiency have restricted their clinical translation (70, 71). Platelets, an emerging biosource for liquid biopsy, are abundant, easily isolated, contain high-quality RNA, and dynamically alter their RNA content in response to external stimuli (72, 73). Although platelets and exosomes remain comparatively understudied, these features underscore their potential for tumor diagnosis and monitoring.

We first performed circRNA sequencing of platelets and matched plasma exosomes from healthy donors (HD) and LUAD patients. The majority of circRNAs in both compartments were exonic, with comparable relative proportions. Each individual yielded 15,000–20,000 known circRNAs in platelets but only ~1,000 in matched plasma exosomes, indicating a markedly higher circRNA content in platelets. These data align with previous reports that circRNAs are enriched in anucleate cells such as platelets, are resistant to nuclease degradation, exhibit greater stability than linear RNAs, and display circRNA/linear-RNA ratios 17- to 188-fold higher than in nucleated cells (74). Collectively, our findings establish that platelet circRNAs not only resemble exosomal circRNAs in composition but also vastly exceed them in abundance, providing a theoretical basis for downstream differential analyses.

Accurate quantification of circRNA expression requires appropriate internal reference genes for normalization. No universal reference gene exists across species or treatments; classical genes such as GAPDH can be unstable under drug treatment (75), and other commonly used genes (PGK1, ACTB, B2M) also fluctuate under different conditions (76, 77). Currently, no consensus reference gene for circRNA quantification has been established. To strengthen biomarker discovery and ensure reliable quantification of platelet circRNAs in LUAD, we screened the most stably expressed circRNAs in platelets from both HD and LUAD patients, and ranked candidates with delta-Ct, geNorm, NormFinder, and BestKeeper algorithms, identifying hsa_circ_0001380 as the optimal reference for qRT-PCR normalization.

During the discovery phase, high-throughput circRNA sequencing identified a panel of circRNAs significantly down-regulated in LUAD patient platelets; qRT-PCR confirmed hsa_circ_0061274 as the most markedly reduced. LUAD is the most common lung-cancer subtype, but early diagnosis remains challenging because symptoms are non-specific and imaging features overlap with benign nodules (78). Among patients undergoing surgical resection for suspected malignancy, 9-55% ultimately prove to have benign disease (79), underscoring the need for peripheral-blood markers that can pre-operatively distinguish benign from early malignant nodules. We therefore expanded an independent validation cohort to evaluate the capacity of hsa_circ_0061274 to discriminate benign pulmonary nodules (BPN) from LUAD, especially stage I disease. qRT-PCR confirmed that platelet hsa_circ_0061274 remained significantly lower in LUAD than in BPN (P = 0.0098), with an AUC of 0.75. Critically, in stage I LUAD tumors ≤3 cm, the circRNA was still significantly reduced (P = 0.019), yielding an AUC of 0.68. While this result highlights its potential for early detection, it also suggests that a single platelet circRNA biomarker may be insufficient to achieve high-accuracy early diagnosis. Therefore, the future application of hsa_circ_0061274 is more likely to be as a component of a multi-marker liquid biopsy panel, complementing biomarkers from other sources to collectively improve the detection rate of early-stage lung adenocarcinoma.

Limitations of the study include its single-center, cross-sectional case–control design, modest sample size, and absence of multi-center independent or prospective longitudinal validation, all of which restrict generalizability. Secondly, the biological function and mechanistic role of hsa_circ_0061274 were not experimentally addressed; its specific role in tumorigenesis remains unknown. Furthermore, the lack of parallel immune profiling precludes any direct inference about the relationship between platelet hsa_circ_0061274 expression and the host anti-tumor immune response. Incorporating such immunological endpoints will be essential to position TEP-derived circRNAs within the evolving landscape of cancer immunodiagnostics. Finally, diagnostic performance was assessed only for the single circRNA, without systematic comparison to conventional tumor markers or other liquid-biopsy analytes such as ctDNA or miRNAs.

In summary, our discovery-phase RNA-seq analysis of this study was based on a small-sample cohort, which may impose limitations when assessing inter-individual variability. However, the key biomarker hsa_circ_0061274 was successfully validated in a larger independent validation cohort, which strengthens the reliability of the core conclusions. We demonstrate that platelet hsa_circ_0061274 stably and efficiently distinguishes LUAD patients from healthy donors and accurately identifies stage I disease, conferring both diagnostic and early-screening value. This is not the first report implicating hsa_circ_0061274 in cancer diagnostics: serum hsa_circ_0061274 is down-regulated in gastric cancer, with the extent of reduction correlating with larger tumor diameter and distant metastasis, and ROC analysis showed that hsa_circ_0061274 alone can separate gastric-cancer patients from healthy controls (60). Thus, down-regulation of hsa_circ_0061274 is not confined to a single tumor type but may represent a pan-cancer phenomenon. Although hsa_circ_0061274 demonstrates promising diagnostic potential, a direct comparison of its performance with established clinical biomarkers or emerging liquid biopsy markers is essential to evaluate its clinical added value. This study was limited by its retrospective design, and complete conventional biomarker data were not available for all patients to enable parallel analysis. Future prospective studies should aim to integrate platelet hsa_circ_0061274 with ctDNA, protein biomarkers, and other modalities to construct a multi-modal diagnostic model, which is expected to further improve the diagnostic accuracy for early-stage lung adenocarcinoma, particularly in the differential diagnosis of pulmonary nodules. Meanwhile, Future studies should aim to integrate platelet circRNA profiling with comprehensive immunophenotyping—including characterization of peripheral immune cell composition and functional status. Such investigations would help position platelet-derived circRNAs not only as diagnostic biomarkers but also as potential windows into the dynamic interplay between the tumor and the host immune system.

## 5 Conclusion

In summary, we provide the first systematic evidence that platelet circRNAs are quantitatively superior to their exosomal counterparts, identify hsa_circ_0001380 as a robust normalizer for platelet circRNA qRT-PCR, and validate platelet hsa_circ_0061274 as a minimally invasive biomarker for LUAD detection and benign–malignant nodule discrimination. These findings position platelet circRNAs as a clinically actionable component of precision oncology workflows.

## Data Availability

The original contributions presented in the study are publicly available. This data can be found here: https://ngdc.cncb.ac.cn/bioproject/, accession number: PRJCA052366.

## Supplementary material

Supplementary Table 1 CirRna count and length 1.

Supplementary Table 2 CirRna count and length 2.

Supplementary Table 3 CirRna count and length 3.

Supplementary Table 4 CirRna count and length 4.

Supplementary Table 5 CirRna count and length 5.

Supplementary Table 6 Control group and LUAD group circRNA sequencing data.

Supplementary Table 7 Relative expression levels of hsa_circ_0061274 in each group.

Supplementary Table 8 Ct value of reference genes.

Supplementary Table 9 The top 10 upregulated and downregulated CircRNAs.
